# Genome-Wide Association Study Reveals Natural Variations Contributing to Drought Resistance in Crops

**DOI:** 10.3389/fpls.2017.01110

**Published:** 2017-06-30

**Authors:** Hongwei Wang, Feng Qin

**Affiliations:** ^1^Agricultural College, Yangtze UniversityJingzhou, China; ^2^Hubei Collaborative Innovation Center for Grain Industry, Yangtze UniversityJingzhou, China; ^3^College of Biological Sciences, China Agricultural UniversityBeijing, China

**Keywords:** GWAS, drought tolerance, gene cloning, crops, natural variation

## Abstract

Crops are often cultivated in regions where they will face environmental adversities; resulting in substantial yield loss which can ultimately lead to food and societal problems. Thus, significant efforts have been made to breed stress tolerant cultivars in an attempt to minimize these problems and to produce more stability with respect to crop yields across broad geographies. Since stress tolerance is a complex and multi-genic trait, advancements with classical breeding approaches have been challenging. On the other hand, molecular breeding, which is based on transgenics, marker-assisted selection and genome editing technologies; holds great promise to enable farmers to better cope with these challenges. However, identification of the key genetic components underlying the trait is critical and will serve as the foundation for future crop genetic improvement. Recently, genome-wide association studies have made significant contributions to facilitate the discovery of natural variation contributing to stress tolerance in crops. From these studies, the identified loci can serve as targets for genomic selection or editing to enable the molecular design of new cultivars. Here, we summarize research progress on this issue and focus on the genetic basis of drought tolerance as revealed by genome-wide association studies and quantitative trait loci mapping. Although many favorable loci have been identified, elucidation of their molecular mechanisms contributing to increased stress tolerance still remains a challenge. Thus, continuous efforts are still required to functionally dissect this complex trait through comprehensive approaches, such as system biological studies. It is expected that proper application of the acquired knowledge will enable the development of stress tolerant cultivars; allowing agricultural production to become more sustainable under dynamic environmental conditions.

## Introduction

In order to meet the demands of the ever-growing human population, it is predicted that world food production will need to double by the year 2050 (Tilman et al., 2002). Unfortunately, crop production is facing severe potential threats from changes within our global climate (Battisti and Naylor, 2009; Boyer et al., 2013). For example, when crops suffer under abiotic stress conditions such as drought, salinity, and temperature extremes, primary losses of crop productivity might occur worldwide with an average yield loss of >50% for major crops (Boyer, 1982). Drought stress dramatically reduces agricultural harvests, resulting in widespread risk of food insecurity and social problems. Thus, given the unpredictable nature of drought and climate variability over the years, breeding crops that are tolerant to abiotic stresses, especially to drought, is one of the most important approaches to maintain or increase crop production (Gill and Tuteja, 2010; Tester and Langridge, 2010).

Due to the complex genetic basis of stress tolerance, classical breeding strategies often fail to meet the needs for stabilizing yield under variable conditions (Tester and Langridge, 2010), although these strategies have significantly impacted the yield potential of crops during the past century, without preexisting knowledge of the exact genetic factors controlling the trait (Collins et al., 2008). On the other hand, molecular breeding approaches, which include transgenic, genome-wide marker-assisted selection and genome editing technologies, are more accurate and rely on clear genetic information of the favorable alleles contributing to the trait. As such, transgenic approaches can insert or create novel alleles beyond those that are available within naturally occurring populations. However, there are limitations for the broad utility of these products due to the requirements for passing environmental safety and regulatory approvals prior to their acceptance for cultivation. In contrast, marker-assisted selection (MAS), which uses DNA markers to track specific chromosomal regions carrying natural favorable alleles in sequential runs of crossing and selection, is free from artificial genetic modification and is widely considered to be natural and safe. Although this approach is time-consuming, the products generated from these breeding strategies have a greatly accelerated path to market. By leveraging the knowledge of biochemistry and genetics, the emerging genome editing technology can efficiently introduce specific mutations into target loci referring and can be considered as transgenic-free. This technology enables direct functional studies of genes of interest in the crop context and provides a suite of new methodologies for crop improvement (Voytas and Gao, 2014; Gao, 2015).

Due to their sessile nature, crops have evolved sophisticated mechanisms to adapt to environmental changes. As a result, it is likely that the functional mechanisms underlying environmental stress responses in plants are probably more advanced and prominent than in animals. The question of how plants survive various environmental stresses is one of the most attractive topics to plant biologists and agronomists. As a result, there is tremendous interest in and demand for enhancing the stress tolerance of crops through biotechnology with the knowledge of how plants react and resist to drought stress. Thus, this review describes advances in natural genetic variations detected for drought tolerance, which may offer genomic selection targets for molecular breeding to accelerate drought-resistance breeding and sheds light into the mechanism of how plants react to stress.

## Qunatitative Trait Loci (QTL) Analysis for Drought Tolerance

**FIGURE 1:**
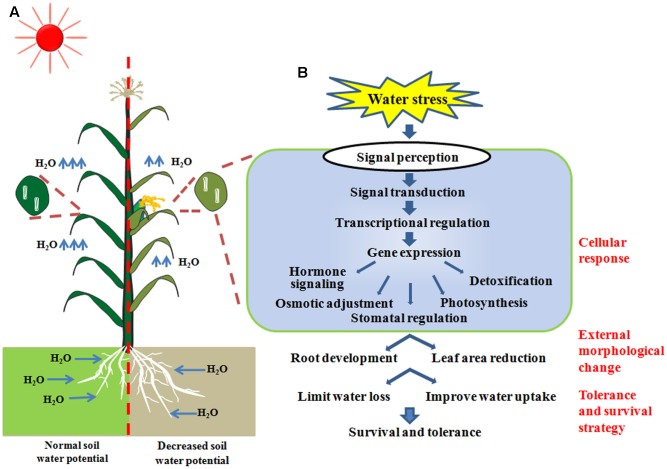
**(A)** The physiological and morphological adaptations of plants grown under water-deficit conditions. **(B)** Cellular response to water stress, including signaling transduction and physiological changes to achieve survival strategies.

The mechanism of how plants respond to drought is sophisticated, which includes processes such as the regulation of genetic and metabolic pathways on molecular, physiological and population levels (Chaves et al., 2003; Izanloo et al., 2008; Xu et al., 2009). Overall, the adaptation to drought can be split into different categories including: ‘drought avoidance’ by escaping the water deficit, owing to inherent developmental distinctions such as early/late flowering time and robust root systems (Geber and Dawson, 1990); ‘water preservation’ of specialized anatomic leaf structures (Schulze, 1986; Jackson et al., 2000) and advanced leaf senescence (Hoffmann and Merila, 1999; Chaves et al., 2003; Sherrard et al., 2009; Maherali et al., 2010); and ‘cellular drought tolerance’ to dehydration (Bartoli et al., 1999). The last aspect is often inferred as ‘drought tolerance’ by researchers specialized in plant abiotic stress response. With respect to the cellular drought tolerance, when soil water potential is decreased, stress related signal transduction networks involving ABA dependent or non-ABA dependent pathways will be stimulated and transduced after these stress signals are perceived by plant cells. Transcription factors, which are master regulators of stress-responsive gene expression, are then subsequently activated. The expression of stress-responsive genes will result in biochemical and physiological processes, including hormone biosynthesis and transport, adjusted osmotic status, photosynthesis, stomatal regulation and detoxification in plant cells. Thus, stress induced morphology changes, including root growth enhancement and leaf area reduction, will result in acquired tolerance and survival strategies (**Figure 1**). All these categories, which can enhance plant survival and growth under the stress, are referred as drought resistance.

In order to dissect the genetic basis of plant drought resistance, forward genetics strategies have been utilized to identify QTLs underlying the trait. However, due to the complexity and poor heritability of the crop yield trait, individual trait components are more frequently identified and characterized due to their better heritability in replicated experiments (Meyer et al., 2007; Riedelsheimer et al., 2012). For example, the trait components regarding maize drought resistance are usually dissected into (1) seedling survival rate under severe water deficits (Liu et al., 2013; Mao et al., 2015; Wang et al., 2016); (2) yield loss which is often indexed by ear length, kernel number per row, and hundred-kernel weight (Lu et al., 2006; Vikram et al., 2012; Yadaw et al., 2013); (3) anthesis and silking interval (ASI), which is an important indicative trait for maize drought tolerance. Drought stress often delays maize ear silk emergence and elongation, but not tassel development and pollen shedding; which leads to a significantly prolonged ASI. Thus, it dramatically reduces pollination efficiency and yields (Lebreton et al., 1995; Ribaut et al., 1996; Tuberosa et al., 2002a; Welcker et al., 2007; Lu et al., 2010). When identifying QTLs for grain yield and ASI across populations and under water stressed and well-watered environments, 68 QTLs were discovered with each physical interval of the QTL consisting of approximately five candidate genes (Semagn et al., 2013).

In addition, ABA is regarded as a stress hormone which can be significantly produced under drought and stimulates the expression of a large number of stress responsive genes and triggers stomatal closure (Bray, 1993; Close, 1996; And and Bartels, 2003). Thus, many studies have focused on the identification of QTLs that could enhance the concentration of ABA under stress in an attempt to better understand drought tolerance (Lebreton et al., 1995; Sanguineti et al., 1999; Landi et al., 2005; Rahman et al., 2011; Iehisa et al., 2014; Barakat et al., 2015). Specially, Landi et al. (2005) found a major maize QTL for L-ABA in bin 2.04 which indicated that the QTL not only enhances yield but also affects root architecture under drought conditions.

In the mean time, root achitechure (e.g., seminal root number; nodal root number; root pulling force; primary root diameter, length and weight; and adventitious seminal root weight) also play an important role in the process of drought resistance (Guingo et al., 1998; Tuberosa et al., 2002b; Gowda et al., 2011; Messmer et al., 2011; Sebastian et al., 2016). Tuberosa and Salvi (2002) reported that a deep root system with higher root density increased the survivability of plants under drought. Furthermore, four QTLs for nodal root angle (qRA), three for root dry weight, two for shoot dry weight, and three for plant leaf area were reported in sorghum; of which nodal root angle QTL presented new opportunities for improving drought adaptation mechanisms (Mace et al., 2012). Specially, introducing *DEEPER ROOTING 1* (*DRO1*) into a shallow-rooting rice cultivar by backcrossing enabled the resulting line to avoid drought via increased root depth; which ultimately resulted in maintenance of high yield performance under drought conditions relative to the recipient cultivar (Uga et al., 2013). The 1 bp deletion in the open reading frame may be the causative site responsible for the differentiating root morphology between IR64 and KP, two rice varieties which differ in root architecture. The discovered allelic variation of *DRO1* not only reveals the possible mechanism of how root morphology is modulated, but also suggests that the control of root architecture will contribute to the drought avoidance of crops. Over the last two decades, many studies have focused on the aforementioned drought resistance trait components in crops (**Table 1**). Thus, our knowledge pertaining to the genetic loci controlling drought resistance has increased substantially.

**Table 1:** Genetic dissction for drought resistance and related traits.

Trait	Species	Mapping population	Closest marker to major locus	Candidate gene	Reference
Components of grain yield	Maize	B73 × Mo17	piol0005	None	Beavis et al., 1994
	Maize	SD3 × SD35	umc76	None	Agrama and Moussa, 1996
	Maize	Ac7643S_5_ × Ac7729/TZSRWS_5_	umc119	None	Ribaut et al., 1997
	Maize	Zong 3 × 87-1	umc1042–bnlg2144	None	Lu et al., 2006
	Maize	X178 × B73	bins 9.03-9.05	None	Hao et al., 2008
	Rice	Basmati334 × Swarna and N22 × MTU1010	RM11943–RM12091	None	Vikram et al., 2012
	Maize	Association mapping panel	PZE-104036909	GRMZM2G125777	Xue et al., 2013
	Rice	IR77298-5-6-18/2 × Sabitri	RM231	None	Yadaw et al., 2013
Anther-silk interval	Maize	SD34 × SD35	umc140	None	Agrama and Moussa, 1996
	Maize	Ac7643S_5_ × Ac7729/TZSRWS_5_	umc174	None	Ribaut et al., 1996
	Maize	X178 × B73	bin 1.03	None	Hao et al., 2008
	Maize	Integrated linkage–LD mapping population	bin2.03	GRMZM2G164400	Lu et al., 2010
	Maize	Association mapping panel	PZE-103179220	GRMZM2G137961	Xue et al., 2013
	Maize	25 MARS populations	19,226,000-22,594,000	112 candidate genes	Semagn et al., 2013
Abscisic acid	Maize	Polj17 × F-2	UMC39b	None	Lebreton et al., 1995
	Maize	Os420 × IABO78	csu133	None	Tuberosa et al., 1998
	Maize	Os420 × IABO78	umc128, csu133, csu109a and umc193d	None	Sanguineti et al., 1999
	Maize	Os420 × IABO78	bin2.04	None	Landi et al., 2005
	Maize	DTP79 × B73	csul29-csu81	None	Rahman et al., 2011
	Maize	Association mapping panel	PZB01403.4	GRMZM2G124260	Setter et al., 2011
	Wheat	Chinese Spring (CS) × Hope5A	Xbarc186-Xgwm617	None	Iehisa et al., 2014
	Wheat	Yecora Rojo × Pavon 76	Wmc161-Wmc96	None	Barakat et al., 2015
Sucrose	Maize	Association mapping panel	PZB02017.1	GRMZM2G173784	Setter et al., 2011
	Maize	Association mapping panel	PZA03635.1	GRMZM2G021044	Setter et al., 2011
Phaseic acid	Maize	Association mapping panel	PZD00027.3	GRMZM2G110153	Setter et al., 2011
	Maize	Association mapping panel	PZD00027.3	GRMZM2G110153	Setter et al., 2011
	Maize	Association mapping panel	PZA03569.2	GRMZM2G125023	Setter et al., 2011
Total sugar	Maize	Association mapping panel	PZA03368.1	GRMZM2G064848	Setter et al., 2011
	Maize	Association mapping panel	PZA03573.1	GRMZM2G092497	Setter et al., 2011
Seedling survival rate	Maize	Association mapping panel	S9_94178074	*ZmVPP1*	Wang et al., 2016
	Maize	Association mapping panel	S10_2680244	*ZmNAC111*	Mao et al., 2015
	Maize	Association mapping panel	S1_201957243	*ZmDREB2.7*	Liu et al., 2013
	Maize	Association mapping panel	allele-338	*ZmPP2C-A10*	Xiang et al., 2017
Root traits	Maize	Lo964 × Lo1016	PGAMCTA205	None	Tuberosa and Salvi, 2002
	Sorghum	B923296 × SC170-6-8	SPb-6287/SPb-9490	None	Mace et al., 2012
	Rice	IR64 × KP	ID07_14 ID07_17	*DRO1*	Uga et al., 2013


## The Development of Population Design, Genotyping, and Variance Components Estimation Approaches for Genome-Wide Association Study (GWAS)

In plants, linkage mapping and genome-wide association study (GWAS) are the two major adopted methods to identify the QTLs for complex traits. The fundamental basis for linkage mapping and association mapping is genetic recombination. Linkage mapping exploits the functional polymorphisms and adjacent markers within families or pedigrees with known ancestry, whereas, association mapping exploits historical and evolutionary recombinations at a natural population level. Populations adopted for linkage mapping are usually derived from a bi-parental cross with a clear ancestry, while a collection of cultivars with unobserved ancestry are often adopted for association mapping (Cockram et al., 2010; Zhao et al., 2011; Hung et al., 2012; Riedelsheimer et al., 2012; Zhang et al., 2015; Zhou et al., 2015). Thus, biased association may occur due to the population structure and imbalanced familial relatedness among cultivars. In order to break the population structure and improve the statistic power for detecting rare variations, the creation and usage of newly designed populations in plants has emerged. In 2008, nested association mapping (NAM) and multi-parent advanced generation inter-cross (MAGIC) were used (Cavanagh et al., 2008; Yu et al., 2008) as methodologies to enable the functional identification of loci of interest. The NAM approach benefits from the historical and recent recombination events with clear population structure, whereas, MAGIC can examine the effect of loci unbiased due to the balanced contributions from all founders. In addition to these approaches, an additional population design is the random open-parents association mapping (ROAM) population which improves genetic resolution and statistical power for detecting rare variations (Pan et al., 2016; Xiao et al., 2016). Collectively, the recently designed populations have demonstrated their capacity to reveal the genetic components underlying complicated traits such as heterosis (Huang et al., 2016), leaf architecture (Tian et al., 2011) and flowering time (Huang et al., 2011).

However, no matter which mapping population is chosen for a study, a large number of molecular markers, which record the level of genetic diversity between two parental lines or amongst different cultivars, are needed for the mapping of QTLs. For example, it was estimated that more than 10 million markers were required for an efficient GWAS in maize, not taking into account of epigenetic variations (Myles et al., 2009). In brief, the development of molecular marker methodologies has undergone three major stages. The earliest type of molecular markers, such as restriction fragment length polymorphisms (Botstein et al., 1980), random amplified polymorphism DNA (Williams et al., 1990), sequence characterized amplified regions (Paran and Michelmore, 1993), cleaved amplified polymorphic sequences (Konieczny and Ausubel, 1993), simple sequence repeats (Litt and Luty, 1989) and amplified fragment length polymorphisms (Vos et al., 1995) rely on DNA hybridization or polymerase chain reaction (PCR) techniques; which are either too expensive or inefficient. As genomic and expression sequence tag information began to accumulate (Adams et al., 1991), the primary molecular markers tools changed from DNA fragment lengths to the single nucleotide polymorphism (SNP) level (Wang et al., 1998); since SNP consists of the largest amount of variations in different genomes (Rafalski, 2002; Zhu et al., 2003). However, SNP detection methods, such as Taqman and molecular beacons, were still time-consuming and expensive (Tapp et al., 2000). Lately, a high-throughput sequencing technology by massively parallel sequencing and image recognition, such as Roche454 FLX Titanium (Thudi et al., 2012), HiSeq2500 (Bentley et al., 2008), Ion Torrent PGM (Rothberg et al., 2011), could obtain more than 100 million nucleotide sequences that could offer millions of SNP markers at a comparatively low cost for a mapping population (Shendure and Ji, 2008; Edwards and Batley, 2010). This is especially true for genotyping-by-sequencing (GBS), which focuses on sequencing the ends of DNA restriction fragments rather than the whole genome. As a result, GBS provides scientists an alternative genotyping method to quickly identify the genomic variations from an unprecedented number of samples; especially for species with large genomes (Altshuler et al., 2000; Narum et al., 2013). Due to the advantages of its efficiency, speed, simplicity and cost-effectiveness (Davey et al., 2011); GBS is an ideal marker acquisition technology (Poland and Rife, 2012). Notably, the GBS method was used for QTL mapping and genome diversity studies (Fu and Peterson, 2011; Deschamps et al., 2012; Fu Y. B. et al., 2013; Lu et al., 2013). For example, 140 million SNP and 200 thousand Insertion/Deletion (InDel) markers amongst 5000 recombinant maize inbred lines (Gore et al., 2009), 680 thousand SNP markers in 2815 maize inbred lines (Romay et al., 2013), 200 thousand SNP markers between wild and cultivated soybeans (Lam et al., 2010) were identified by GBS. Thus, high-throughput sequencing technology undoubtedly provides a revolutionary tool for offering a large amount of whole genome-coverage genetic markers for gene mapping either through linkage and/or association analyses to dissect the genetic loci underlying any interested traits in crops.

In addition to the sufficient amount of molecular markers, the fast and accurate variance component estimation method is also one of the prerequisites to perform a GWAS. In the early stage of GWAS, the general linear model was employed which included genome control (Henderson, 1975), family test (Abecasis et al., 2000) and structural correlation (Pritchard et al., 2000) to control possible false positive associations. Later, Yu et al. (2006) proposed a method based on a mixed linear model (MLM) for better control of the population structure and the imbalance correlation among various materials; of which the population structure (Pritchard et al., 2000) and the familial relatedness among the different genotypes were treated as fixed effects and random effects, respectively. However, no matter which statistical method is applied, a large amount of samples is needed to obtain sufficient statistical power (Balding, 2006). This is especially important to detect the associations of a complex trait to loci with small effects (Buckler et al., 2009). Since the computational burden is proportional to the cube of the sample number fitted as random effects, massive computational time is demanded by the MLM. Especially, when associations are analyzed for a large number of markers with the phenotypic information from thousands of individuals, heavy computational burden occurs. In order to solve this problem, the sire model was first used in animal association studies to enhance the computational speed by reducing random effects (Henderson, 1975; Thompson, 1979; Quaas and Pollak, 1980; Pollak and Quaas, 1983). After that, genome-wide rapid association analysis using mixed model and regression (GRAMMER) built in GenABLE software was developed to approximately estimate the random effects via a two-step residuals approach. It estimates the residuals from the linear mixed model by removing the marker effects first, and then the residuals are treated as a phenotype for each marker under a standard linear regression model (Aulchenko et al., 2007). This method significantly reduced the computational time for each marker association calculation. Unlike the approximate estimation, efficient mixed-model association (EMMA) matrix is a method for an accurate estimation of variance component, including the genetic variance and residual variance, which can speed up the iterative process (Kang et al., 2008). However, for a GWAS of a population consisting of a few thousand individuals and a half million SNPs, it is probable that several years of central processing unit time would be necessary to complete the computing when using the EMMA method (Kang et al., 2010; Zhang et al., 2010). Population parameters previously determined (P3D) (Zhang et al., 2010) and EMMA eXpedited (EMMAX) (Kang et al., 2010) were the other two complex approximate estimation methods to reduce the computational processing. The two calculation methods made it possible for a personal desktop computer to perform GWAS; which adopts 1000 samples with 500 thousand markers or more. Although in most cases, the approximate variance estimation method would generate a nearly identical estimation as compared to the accurate method (Kang et al., 2010; Zhang et al., 2010). The accuracy of approximate estimation would be unknown in the absence of accurate estimation for variance components. Thus, factored spectrally transformed linear mixed models (FaST-LMM) (Lippert et al., 2011) and genome-wide efficient mixed-model association (GEMMA) (Zhou and Stephens, 2012) were developed. These approaches had advantages to directly estimate variance components including fixed effects in MLM and reduce the calculation burden for the single maker analysis. Benefitting from continuous efforts, with the massively reduced computational time and effective statistics method, the GWAS strategy became computationally practical the enable the genetic dissection for a trait of interest.

## GWAS Facilitates QTL Cloning for Drought Tolerance

Although several QTLs for drought resistance have been identified through traditional linkage analysis, to date, no QTLs responsible for drought resistance have been cloned; despite the reports of their mapping information (Hao et al., 2009; Messmer et al., 2009; Almeida et al., 2013; Semagn et al., 2013). In order to clone a stress resistant QTL through traditional segregation and linkage analysis, not only do the different recombinants in the identified QTL region need to be screened, but the resistance of their progenies also need to be phenotyped and compared to determine which genetic region co-segregates with the resistance. In order to narrow down the QTL region to candidate genes, it usually takes several rounds of the fine mapping step; which makes QTL cloning time-consuming and laborious. As a result, the identification of the genetic components underlying drought resistance has been considered and proven to be very challenging. Nevertheless, owing to the advantage of a rapid life cycle and less space requirements for the cultivation of the model plant compared with crops; significant achievements have been made in *Arabidopsis thaliana* to study the molecular mechanisms for drought tolerance. Although, the genetic basis of quantitative variation for drought resistance in crops remains poorly understood, a complex gene network involving various kinds of genes has been conceived (Li et al., 2005; Qin et al., 2011; Osakabe et al., 2014).

Based on linkage disequilibrium (LD), the GWAS method enabled researchers to greatly increase the knowledge base of the genetic architecture and it also facilitated the cloning of genes underlying complex traits in crops. For example, the genetic basis of heterosis (Huang et al., 2016) and some important agronomic traits (Cockram et al., 2010; Tian et al., 2011; Sukumaran et al., 2014; Zhang et al., 2015; Si et al., 2016) were investigated through GWAS in a large amount of collected or constructed populations. However, it was estimated that for self-pollinating species, such as rice and soybean, the LD decay of cultivated rice had a long-range from about 100 kb to over 200 kb (McNally et al., 2009; Huang et al., 2010); whereas those of soybean landraces and improved cultivars were approximately 83 and 133 kb, respectively (Zhou et al., 2015). Whereas, for maize, which is an out-pollination species, the LD decay was estimated to be ≤2 kb among 26 founder genotypes of a nested association mapping population (Gore et al., 2009); whereas the LD decay was ∼500 bp in a maize natural variation population (Fu J. J. et al., 2013). Therefore, the rapid LD decay in the maize genome with above 1 million high-quality SNPs (Fu J. J. et al., 2013; Li et al., 2013) could make the resolution of GWAS reach the single gene level (Fu J. J. et al., 2013; Li et al., 2013; Wang et al., 2016). As a result, GWAS facilitated the gene cloning of *ZmCCT* which controls maize photoperiod sensitivity (Hung et al., 2012; Yang et al., 2013) and the investigatigation of evolutionary *ZmWAK* locus which controls the resistance to *Sporisorium reilianum*, a soil-borne fungus causing head smut disease in maize (Zuo et al., 2015).

Drought resistance is a complex and intrinsic trait, and the improvement of plant drought resistance through molecular breeding strategy is an ever growing hotspot for both basic and applied research areas. By leveraging knowledge gained from model plants, plant drought tolerance has been enhanced by manipulating the expression of drought-responsive genes (Li et al., 2005; Qin et al., 2011; Osakabe et al., 2014). In crops, however, limited allelic variation underlying drought tolerance has been identified. Recently, GWAS has contributed to the identification of favorable alleles for drought tolerance, especially in maize. Setter et al. (2011) reported that a candidate gene association analysis identified loci involved in the accumulation of carbohydrates and ABA metabolites under drought; of which eight candidate SNPs were confirmed to be significantly associated with the tolerance. Later, each genotype of this association panel was crossed with a common tester (CML312) and the generated F_1_ plants were phenotyped for nine traits under well-watered and water-stressed conditions in seven environments. As a result of this analysis, an additional 42 associated SNPs were identified (Xue et al., 2013). Liu et al. (2013) analyzed all the functional (Dehydration Responsive Element Binding protein) *DREB* genes in maize and examined their associations with the natural variation in drought tolerance among 368 maize varieties collected from tropical/subtropical and temperate regions (Fu J. J. et al., 2013; Li et al., 2013). A significant association of the natural variation in *ZmDREB2.7* gene with drought tolerance was detected which locates in the gene promoter region and most likely enables an early induction of stress responsive gene expression. Thirunavukkarasu et al. (2014) reported that by using 240 accessions of subtropical maize with a high density marker set, 61 significant SNPs were identified under water stress condition; 48% of which were stress tolerance related genes. Moreover, the genetic basis of drought resistance in *Hordeum vulgare*, *Cicer arietinum*, *A. thaliana*, *Medicago truncatula* were also investigated by GWAS (Thudi et al., 2014; Verslues et al., 2014; Kang et al., 2015; Wehner et al., 2015). Recently, combined data from 15 bi-parental populations of maize were developed under the Water-Efficient Maize for Africa (WEMA) project to identify drought resistance genes by GWAS. The study identified several hundred genetic variants that are associated with plant height and flowering time under various water deficit conditions (Wallace et al., 2016). By adopting a global maize diversity panel (Yang et al., 2010; Fu J. J. et al., 2013; Li et al., 2013), Wang et al. (2016) reported a GWAS of maize drought resistance at the seedling stage and identified 83 genetic variants, which were resolved to 42 candidate genes. The peak GWAS signal overlapped with a previously reported drought related QTL9.3 (Semagn et al., 2013), and is directly located in the *ZmVPP1* gene on Chromosome 9 which encodes a vacuolar-type H^+^-pyrophosphatase (H^+^-PPase). The *ZmVPP1* gene is a homolog of *AVP1* which encodes an *Arabidopsis* H^+^-PPase. The *AVP1* gene was shown to confer drought and salt resistance when over-expressed in *Arabidopsis* (Gaxiola et al., 2001). Yeast and *E. coli* H^+^-pyrophosphatases can establish a proton gradient across the vacuolar membrane by means of pyrophosphate (PPi) hydrolysis (Maeshima, 2000; Rea and Poole, 2003) and acidify the vacuole (Lin et al., 2012). Other studies also found that transgenic plants expressing the H^+^-PPase from *Arabidopsis* or other species developed larger root systems and were more resistant to salt, drought stress, and phosphorous deficiency (Gaxiola et al., 2001; Park et al., 2005; Zhao et al., 2006; Li et al., 2008; Lv et al., 2008; Bao et al., 2009; Pasapula et al., 2011; Pei et al., 2012; Schilling et al., 2014). The GWAS study demonstrated that the natural variation in *ZmVPP1*, had the most significant impact to the drought resistance of young seedlings on a genome-wide scale. In the tolerant allele of *ZmVPP1* containing three MYB recognition sequences (WAACCA, W: A or T), a 366-bp insertion was found as the causative variation that conferred the drought inducible expression of *ZmVPP1* (Wang et al., 2016). Moreover, an additional natural variation in *ZmNAC111* located on maize Chromosome 10, which encodes a NAM, ATAF, CUC2 (NAC) type transcription factor, was also found to contribute to the tolerance. ZmNAC111 belongs to a plant-specific transcription factor superfamily which is comprised of members that participate in various biological processes, including plant development, stress response, leaf senescence, and ion re-location (Uauy et al., 2006; Jeong et al., 2010; Nakashima et al., 2012; Liang et al., 2014). An 82-bp miniature inverted-repeat transposable element, forming a stem-loop structure, inserts in the gene promoter. It represses the gene expression most likely through RNA-directed DNA and histone methylation (Mao et al., 2015). Further transgenic data suggested that the biological function of *ZmNAC111* and *ZmVPP1* were different and are most likely functionally involved in the reduction of water loss under water deficit or the enhancement of water uptake and leaf photosynthesis (Mao et al., 2015; Wang et al., 2016). Notably, in *Arabidopsis* it is found that clade A protein phosphatase 2C genes (*PP2C-As*) negatively function in ABA signaling and plant drought response (Ma et al., 2009; Park et al., 2009). In order to find the natural variations of *ZmPP2C-As* that are directly associated with maize drought tolerance, a candidate gene association analysis was conducted. Among this gene family, *ZmPP2C-A10* was found to be tightly associated with drought tolerance. Furthermore, a causal natural variation of this gene was identified, lacking an endoplasmic reticulum stress response element (ERSE) in the gene promoter due to a 14-bp deletion, could lead to increased plant drought tolerance (Xiang et al., 2017).

Furthermore, the adoption of global natural varieties which originate from different regions and periods of time, GWAS can infer the trend of evolution and/or the artificial selection of an important trait. Crops have been subjected to cultivation and extensive selection for grain productivity and quality to meet with human demand. For example, the *ZmWAK* locus, which conferred resistance to head smut, was lost from the teosinte wild ancestry during the domestication of maize (Zuo et al., 2015). Thus, a GWAS population consisting of the ancestral varieties of a species and those from adverse environments would be of great value to identify genetic loci contributing to the stress tolerance or help to understand the domestication history of an important trait by utilization of a superior allele (Hung et al., 2012; Yang et al., 2013; Zhou et al., 2015).

## Network Analysis Offers A New Tool to Reveal the Mechanism for Drought Tolerance

Despite the fact that stress responsive genes are often studied in a one by one manner to undercover their roles in stress tolerance, coordinated expression of related genes has also been identified. For example, microarray and RNA gel blot analyses confirmed that a drought-inducible transcription factor (DREB2A) can regulate the expression of many stress-responsive genes (Sakuma et al., 2006). Findings such as this support the strategy for enhancing stress tolerance by manipulating a single regulatory gene and systematically altering the expression of a large number of genes involved in the drought-responsive network. Despite a large number of genes that are involved in the co-expression network, based on the integrative large scale data acquisition and analysis, systems biology offers a new and integrated tool to dissect the network. Sulpice et al. (2010) found that by profiling maximum catalytic activities of 37 enzymes from central metabolism, a matrix was generated to investigate species-wide connectivity among metabolites, enzymes, and biomass. The results showed that biomass can be predicted by two independent integrative metabolic biomarkers that could result in the preferential investment in photosynthetic machinery and optimization of carbon use. The genetic loci controlling differential gene expression in maize kernels were investigated through GWAS, which revealed a comprehensive gene regulatory network consisting of 31 zein and 16 key kernel genes (Fu J. J. et al., 2013). A comprehensive study of maize kernel metabolites, in relation to genetic variations and gene expressions, identified biomarkers associated with kernel weight which may facilitate the genetic improvement of maize (Wen et al., 2014). Undoubtedly, stress resistance consists of a combination of dynamic networks that contain many correlated genes. The potential for the expression of a specific gene to enhance stress tolerance or not depends on multiple factors such as its position, branch point, direction, and redundant reactions in the network. Recently, network analysis has provided a framework for understanding and modulating plant responses to salt stress and abscisic acid application, through chromatin immuno-precipitation sequencing. Genome-wide targets of 21 ABA-related transcription factors were identified and a comprehensive regulatory network was constructed. Moreover, a new family of transcriptional regulators was discovered to be functionally involved in ABA and salt responsiveness, and shown to modulate plant tolerance to osmotic stress in *Arabidopsis* (Song et al., 2016). Taken together, knowledge derived from network analyses will provide us with new strategies to cope with the ongoing challenges of drought stress.

## Stacking or Combining Favorable Alleles to Improve Drought Tolerance

The key to meet the ever-growing demand for crops yield, and to relieve the threat on crop productivity imposed by environmental stress, is to cultivate high-yield and high-resistance varieties. As a result, it is very important that breeders can have access to tools and technologies that will enable them to improve their selection efficiency. Molecular breeding, which includes MAS, genome editing, genome-wide selection and transgenics, is based on the knowledge of QTL/gene mapping and cloning and is considered as an innovative approach for precision breeding in the 21st century to meet growing demand for food sources (Collard and Mackill, 2008; Voytas and Gao, 2014; Gao, 2015). A favorable allele from wild soybean was used to increase seed protein content in soybean by MAS (Sebolt et al., 2000). Merchuk-Ovnat et al. (2016) generated a near isogenic line (NIL-7A-B-2) by introgressing a drought-related QTL on chromosome 7A from wild emmer wheat into a cultivated wheat variety; resulting in enhanced grain yield and biomass production across environments. Notably, by using the (CRISPR)-Cas9 genome editing technology (Cong et al., 2013; Mali et al., 2013), targeted mutations were successfully introduced into the three homoeoalleles encoding MILDEW-RESISTANCE LOCUS (MLO) proteins; which conferred heritable resistance to powdery mildew in hexaploid bread wheat (Wang et al., 2014). The reported natural allele variation of *DRO1* (Uga et al., 2013), *ZmDREB2.7* (Liu et al., 2013), *ZmNAC111* (Mao et al., 2015), and *ZmVPP1* (Wang et al., 2016) shed novel insight into how natural variation factors into improving crop drought tolerance or resistance. Moreover, the cumulative effects of multiple quantitative resistance loci could be exploited to produce high tolerance (Miedaner et al., 2006; Richardson et al., 2006). Thus, it is plausible that several favorable alleles, not restricted to *ZmDREB2.7*, *ZmNAC111*, and *ZmVPP1*, can be stacked to improve drought tolerance in maize and other crops by marker-assisted selection or genome editing technology.

## Perspectives

Global climate change threatens crop production worldwide. Unexpected changes in weather patterns, such as high temperature and drought have dramatically affected crop yield which in turn could result in food and societal crises. It is plausible that molecular breeding strategies may speed up the traditional breeding processes to increase stress resistance in cultivars; enabling them to better cope with the changing environment. Along with the development of high-throughput DNA sequencing technology, whole genome covered markers can be produced more cost-effectively with unprecedented increases of accuracy. Meanwhile, the advances of statistical analyses for quantitative genetics provide new methodologies to dissect the genetic basis of complex traits. It can be anticipated that systematic network analyses, consisting of genomics, transcriptomics, proteomics, metabolomics, and phenomics, will provide integrative information for increase our understanding of the balance between stress response and grain yield and quality. Undoubtedly, this acquired knowledge will be of great value to put us in a better position to enable the precise molecular design of new cultivars with desired stress resistance and yields.

## Author Contributions

HW wrote the manuscript. FQ designed and revised the manuscript.

## Conflict of Interest Statement

The authors declare that the research was conducted in the absence of any commercial or financial relationships that could be construed as a potential conflict of interest.
